# Comparative evaluation of postoperative pain and periapical healing after root canal treatment using three different base endodontic sealers – A randomized control clinical trial

**DOI:** 10.4317/jced.59034

**Published:** 2022-02-01

**Authors:** Akshay Khandelwal, Jerry Jose, Kavalipurapu-Venkata Teja, Ajitha Palanivelu

**Affiliations:** 1 BDS MDS. Department of Conservative Dentistry and Endodontics, Saveetha Dental College, Saveetha Institute of Medical and Technical Sciences, Saveetha University, Chennai, Tamil Nadu, India

## Abstract

**Background:**

The aim of the present study was to evaluate and compare the postoperative pain and periapical healing after root canal treatment using three different base endodontic sealers.

**Material and Methods:**

Primary root canal treatment was initiated in 63 patients diagnosed with necrotic pulp and apical periodontitis, cleaning and shaping was completed in two visit and different base endodontic sealers were used for obturation were selected based on the random allocation of the participants to the following groups; Tubli-Seal, AH Plus and BioRoot RCS. Postoperative pain was recorded by using 100 mm visual analog scale at 24 h, 48 h, 72 h and 7 d after obturation. Digital periapical radiographic evaluation was done to assess rate of periapical healing at baseline, 1, 3 and 6 months. Statistical analysis was done using Kruskal Wallis test and one-way ANOVA.

**Results:**

The mean difference in the size of periapical lesions for Tubli-Seal (6.27, 13.41), AH Plus (3.86, 9.80) and BioRoot RCS (4.05, 10.22) at 3 months and 6 months respectively. The mean pain scores at 24 h for Tubli-Seal (17.94 ± 11.35), AH Plus (11.57 ± 11.18), BioRoot RCS (4.73 ± 7.72). At 48 h, Tubli-Seal (5.26 ± 9.04), AH Plus (1.57 ± 3.74) and BioRoot RCS (1.57 ± 3.74) respectively. The mean pain score at 72 h for Tubli-Seal was 2.63 ± 7.33 whereas none of the patients had reported pain in AH Plus and BioRoot RCS group. None of the patients had pain after 7 d of treatment.

**Conclusions:**

BioRoot RCS showed less postoperative pain compared to AH Plus and Tubli-Seal and showed better periapical healing compared to AH Plus and Tubli-Seal at 3 and 6 months intervals respectively.

** Key words:**BioRoot RCS, root canal obturation, root canal sealers, periapical periodontitis, Periapical healing, postoperative pain.

## Introduction

Apical periodontitis is an inflammatory lesion around the periapical region and can have significant influence on the endodontic treatment prognosis. Teeth with apical periodontitis are considered to be an entombment of various pathological flora which can influence the success rate of the treatment (1). The aim of endodontic treatment in such scenarios is to maintain an adequate biological environment allowing physiological healing to occur. This is achieved by the complete disinfection of root canal system using root canal irrigants such as sodium hypochlorite (NaOCl) and ethylenediaminetetraacetic acid (EDTA) which actively act on the organic and inorganic portion of the smear layer matrix seen to be a reservoir for various microorganisms (2). In regard to these cases the primary success of conventional endodontic treatment depends on the elimination of organisms responsible for causing periapical pathology. Root canal obturation plays a crucial role in endodontic therapy since it reduces bacterial contamination by preventing coronal leakage and by sealing the apex from periapical tissue fluids and it also entombs the remaining microbes in the canal preventing further disease (3).

Periapical healing is seen to be structural and functional replacement of the bone considered to be an intricate interplay between the osteoclasts and osteoblasts allowing the bone formation to occur seen to be influenced by the host’s intrinsic and extrinsic mechanisms (4). Root canal sealer is a local factor which interferes with the healing of periapical tissues by leaching through the apical foramen and lateral canals (5). Based on the composition of root canal sealers such as zinc oxide-eugenol formulations, calcium hydroxide sealers, glass ionomer sealers, resin based (epoxy resin or methacrylate resin) sealers and recently introduced bioceramic based sealers, the periapical healing can be influenced by changing the rate of bone deposition as well as creating an enhanced environment for remineralization to occur (6).

Postoperative pain is considered to be another significant clinical outcome exhibiting a multifactorial response to treatment related factors such as maintaining the working length to the apical constriction, finishing the endodontic treatment in single visit or multiple visit, instrumentation technique and the type of endodontic sealer used for obturation (7). Postoperative pain usually ranges from 3% to 58% based on the individual’s pain perseverance and stimulus (9). Such pain occurrence is mainly due to mechanical, chemical or microbial injury to the periapical tissues. Root canal sealers can play a crucial role in this regard by coming in contact with the periapical tissues through apical foramen and lateral canals causing a localized inflammation with a direct influence on the degree of inflammation based on the composition of the sealer in turn influencing postoperative pain levels (8).

In regard to all this prior discussed factors, the present study aimed to address both these issues by conducting a comparative evaluation of the incidence and intensity of postoperative pain and post obturation healing of periapical lesions after primary root canal treatment using representatives of different base endodontic sealers such as zinc oxide eugenol sealer (Tubli-Seal), resin-based sealer (AH Plus) and bioceramic sealer (BioRoot RCS) in patients with diagnosed with necrotic pulp and apical periodontitis. The null hypothesis was considered that there was no significant difference in the incidence and intensity of postoperative pain and periapical healing after root canal treatment using BioRoot RCS, AH Plus and Tubli-Seal as endodontic sealers.

## Material and Methods

-Ethical clearance and protocol registration

The present study adhered to the Consolidated Standards of Reporting Trials (CONSORT) statement of reporting (Additional file 1). Ethical approval was obtained from the Institutional Review Board (SRB/SDMDS11/17ODS/09). The protocol for the present clinical trial was registered with the clinical trials registry – India (CTRI) with registration number (CTRI/2018/10/015919) before the clinical trial began. All the patients consented to an informed consent form discussing the details of this trial as well as benefits and risks of the study.

-Trial Design

This study was conducted under a university setting which followed a double blinded parallel randomized clinical trial design with allocation ratio of 1:1 in which the patient and the assessors were blinded from the process. The treating operator could not be blinded since treatment protocol with different endodontic sealers could not be concealed from the operator used in the present study.

-Sample size calculation

A priori sample size calculation was done using G*Power 3.1.2 software based on a previously published study (9). Using a one-way ANOVA fixed effects omnibus model (α = 0.05, 1-ß = 0.95, f = 0.05), the minimum sample size calculated was 18 per group. Expecting an attrition of the sample during follow up, the sample size was adjusted by 10% to 63, 21 per group.

-Study groups

For the present study, 3 groups were taken which were representatives of different base endodontic sealers; Group 1: ZOE based sealer (Tubli-Seal, Sybron Endo, Romulus, MI), Group 2: epoxy resin based sealer (AH Plus, Dentsply DeTrey, Konstanz, Germany), Group 3: Bioceramic based sealer (BioRoot RCS, Septodont, USA).

-Inclusion/Exclusion criteria

Participants undergoing endodontic therapy in maxillary anterior teeth within the age group of 18-60 years categorized under American society of Anesthesiologists (ASA) - 1 giving a tooth diagnosis of necrotic pulp with chronic apical periodontitis confirmed using sensibility test [cold test (1, 1, 2-tetrafluoroethane. Hygienic Endo-Ice Green [Endo-Ice]; Coltene Whaledent, Cuyahoga Falls, OH) and electrical pulp testing (Analytic Technology Pulp Tester, Analytic Technology, Redmond, Wash)] with a periapical index (PAI) score of 2 or more diagnosed using digital radiography as well as a patient’s VAS Score of 30 mm and above were taken into inclusion criteria for the present study since the present study aimed to evaluate two clinical parameters; postendodontic pain and periapical healing. Patients classified other than ASA -1, immature permanent tooth, tooth exhibiting endodontic-periodontic lesions, dystrophic calcifications within the tooth as well as more than 20° curvature, pregnant or lactating women, root fracture cases, patients who consumed analgesics before 12-24 h prior to the primary root canal treatment were excluded from the present study.

-Randomization

Computer generated random Table of numbers was conducted using an online service (random.org) was used for the randomization process to assign the participants to different study groups. Block randomization method was advocated using the SNOSE (sequentially numbered, opaque, sealed envelopes) method for allocation concealment. Two experienced endodontists (V.T, J.J) who were not involved in the treatment process assessed these periapical radiographs and came to consensus regarding the scoring outcome for the size of the lesion. The endodontists prior to evaluation of the radiographs were calibrated based on a set of 100 predetermined radiographs as a calibration exercise and had come to an agreement based on consensus with a 0.70 cohen kappa statistic (*p* < 0.05). The calibration process was continued in a timeframe of 4 d, 7d, 21 d in order to maintain the calibration between the evaluators prior to the start of trial. In case of disagreement, a third specialist who had sufficient experience for more than 10 years on interpreting radiographs was consulted to achieve an agreement. A piece of paper containing the randomized group number was sealed in the dark-colored envelope containing the respective serial and treatment protocol for only the sealer groups prepared by the respective third person (A.P). Study numbers were sequentially assigned to patients by an individual not related to the present study. The envelope was opened once the intervention was assigned. Respective treatment was carried out based on the group assigned in the paper by a single operator (A.K) conducted the treatment.

-Treatment protocol

All the study groups underwent the same protocol. Prior to the treatment, a digital radiographic evaluation was conducted using a customized grid with paralleling technique. Lesion sizes which gave a PAI score 2 or more were recruited for the present study. The treatment protocol was explained to all the participants and an informed consent was obtained. A total of 63 patients were recruited in this study, who fulfilled the above mentioned selection criteria. The teeth were isolated using a rubber dam using a single tooth isolation technique, caries excavation was conducted and a pre-endodontic build up was done using composite resin (3M Filtek, 3M ESPE, USA), if required. Access cavity preparation was conducted using Endo Access Kit (Dentsply Maillefer, Ballaigues, Switzerland) followed by which debridement of pulp chamber contents was done using a spoon excavator and 3% sodium hypochlorite (NaOCl) (Prime dental products, Thane, India). An ISO size 10 K-File (Mani Corp. Japan) was used to obtain for initial patency filing and working length was recorded using an electronic apex locator (Root ZX II, J. Morita, MFG. Corp. Kyoto, Japan) such that it was measured at 0.5 mm short of the apical canal terminus (‘0’ reading). The confirmation of the working length was done by using digital periapical radiograph.

The canals were shaped using the Protaper Gold rotary system (PTG, Dentsply Maillefer, Ballaigues, Switzerland) for all the teeth. The apical preparation was carried out by using ISO stainless steel hand K files (Dentsply Sirona, Ballaigues, Switzerland) starting with the file which was initially binded till the working length, the final instrumentation was carried out 3 sizes larger than the first file followed by which a similar taper instrumentation technique using Protaper Gold till the working length. During the instrumentation process, 3% NaOCl (Prime Dental, Thane, India) was used during each cycle of instrumentation and the canal patency was maintained by passing ISO 15 No. K file approximately 1 mm beyond the determined working length after each instrumentation cycle. In order to effectively remove the smear layer, an irrigation of 17% EDTA (Anabond Stedman, Kanchipuram, India) followed by a final irrigation of 3% NaOCl was conducted. All the irrigation process was conducted using a 30-gauge double side vented needle (Neoendo, Orikam Healthcare, India) and 2 ml syringe barrel (Dispovan, India). During the irrigation process each cycle was intermittently activated using sonic activation for a period of 60 s (EndoActivator, Dentsply Sirona, USA).

Post biomechanical preparation procedure, the canals were dried using sterile paper points according to corresponding taper and freshly mixed calcium hydroxide paste was placed into the prepared canals using a lentulospiral (Dentsply Sirona, USA) followed by which a temporary seal was done using intermediate restorative cement (IRM, Dentsply Sirona, USA). The patients were recalled after a week and patients who were asymptomatic exhibiting a VAS Score of 0 and dry canals on evaluation with sterile paper points after removal of the intra canal medicament were further treated. The teeth were obturated according to the randomly allocated groups, Group 1: Tubli-Seal; Group 2: AH Plus; Group 3: BioRoot RCS.

Manufacturer’s instructions were followed for mixing the respective sealer on a sterile glass slab. The apical extent of the master cone was confirmed radiographically by digital radiograph. The canals were dried using sterile paper points and were coated with the sealer using lentulospiral (Dentsply Canada, Woodbridge, Canada) in a slow speed handpiece (NSK, Tochigi, Japan) followed by which the obturation process was performed with respective sealers using the lateral compaction technique.

Post treatment occlusal reduction of 1 mm was done in all the treated teeth and permanent restorations was done with composite resin (Filtek Z 350, 3M ESPE, USA) and the periapical healing and postoperative pain was assessed. All the clinical procedures were performed by one operator of similar endodontic clinical experience. A partial coverage or a full coverage prosthetic management was done for all the teeth as indicated.

-Outcome assessment

Periapical healing assessment

All the digital radiograph was carried out using parallel cone technique using RVG sensor (Carestream Dental LLC, Atlanta, GA) with the help of a sensor positioning system (Bluedent, India) and was evaluated for baseline data. The data was analyzed by two experienced endodontists (V.T, J.J) who were not involved in the treatment protocol such that prior images were seen so the interobserver agreement was seen at 0.90 using Cohen kappa (*p*<0.05) in regard to periapical diagnosis. The presence or absence of sealer extrusion was also noted. The size of the periapical lesion was calculated with the help of a grid, X-ray mesh gauge (Bluedent, India) such that the entire proximity of the periapical lesion was covered under the mesh gauge and the same observers (V.T, J.J) confirmed the size of the lesion at repeated intervals and mean scores were taken. Subsequent radiographs were taken for each patient at 1, 3 and 6 months using and evaluated using the similar technique. In an event of sealer extrusion during the treatment procedure, the rate of sealer extrusion and pain was assessed separately.

Post treatment pain reduction assessment

All the patients were handed over a pain diary form with visual analogue scale (VAS) consisting of a 100 mm line divided into 10 equal parts from 0 indicating no pain to 100 indicating extremely severe pain. This provided a range of score from 0-100, score 1-29 was graded as mild pain, 30-69 was regarded as moderate pain and 70-100 were regarded as severe pain. The patients were asked to record at 24 h, 48 h, 72 h, and 7 d after treatment followed by which the patients were recalled to give the diary to the investigators. In case of consumption of analgesics, the type and quantity after treatment was also recorded.

-Statistical analysis

Data was entered in Microsoft excel spreadsheet and analyzed using SPSS software (ver. 22, IBM Corporation, Armonk. USA). The normality tests Kolmogorov-Smirnov and Shapiro-Wilk’s test results reveal that all variables did not follow normal distribution. Therefore, a non-parametric test was applied to analyze the data. Chi Square test was used to assess the difference in the extrusion rates among the groups. Mann Whitney U Test was used to assess the differences in the mean pain scores at different time intervals based on extrusion. Kruskal Wallis test was used to assess the differences in mean periapical lesion area and pain score between the various groups. Friedman’s Two-way Analysis of Variance was used to assess the difference between the mean area of periapical lesions measured within each group at different time intervals. For the test, a *p* value of less than 0.05 is to be considered a significance level.

## Results

**Figure 1:**
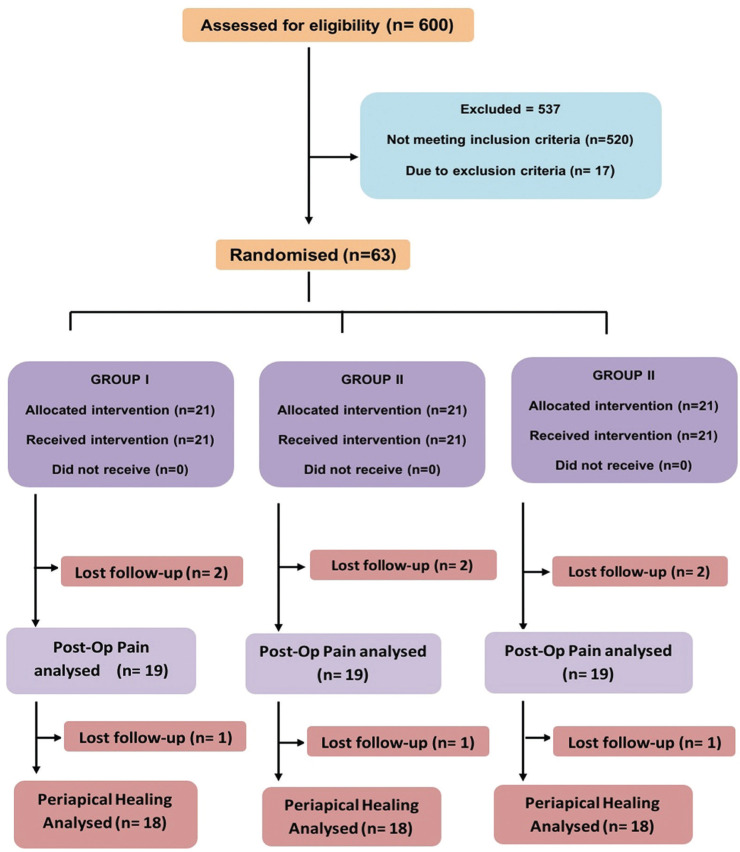
CONSORT flowchart showing the design of the present study with overview of the different treatment protocols, loss of patients to follow up and periapical healing analysis.

600 patients were checked for eligibility over a period of six months, out of which 520 did not meet the inclusion criteria and 17 were excluded because of exclusion criteria; 1 patient was pregnant, 5 had history of diabetes mellitus and 11 patients refused to participate. A total of 63 patients were available for further analysis (Fig. 1). 6 participants had not reported for follow up appointments and hence were excluded. In conclusion data from a total of 57 patients there was a further loss of follow up for 3 patients at 1 week after the treatment interval making it 9 loss of patients (14.3%) making a total of 54 participants data were collected and subjected to analysis. In regard to patient characteristics (Table 1), there was no statistical difference between the age (*p*=0.909) whereas there was a correlation seen based on the gender of the patients (*p*=0.358).

**Table 1:**
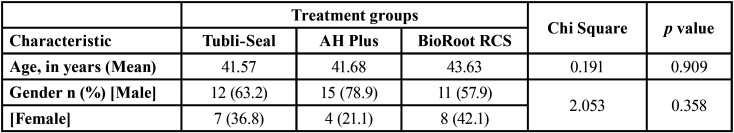
Characteristics of the included participants based on age and gender. No significant difference (*p*>0.05) was seen based on gender and age for the assessed groups.

Table 2 denotes the difference in the mean area of the periapical lesion from baseline to 6 months, there was no difference in the mean area of the periapical lesion at baseline interval, and reduction of size of lesion was seen at 3 and 6 months for all the test groups (*p*=0.001). The mean difference in the size of periapical lesions from baseline to 1 month were Tubli-Seal (0.833) (*p*=0.137), AH Plus (1.08) (*p*=0.09) and BioRoot RCS (1.41) (*p*=0.029); from baseline to 3 months for Tubli-Seal (4.05) (*p*=0.000), AH Plus (3.86) (*p*=0.000) and BioRoot RCS (6.27) (*p*=0.000); from baseline to 6 months, Tubli-Seal (10.22) (*p*=0.000), AH Plus (9.80) (*p*=0.000) and BioRoot RCS (13.41) (*p*=0.000) respectively and shows a significant difference in the reduction of mean area of periapical lesion at 3 months and 6 months in all the three groups.

**Table 2:**
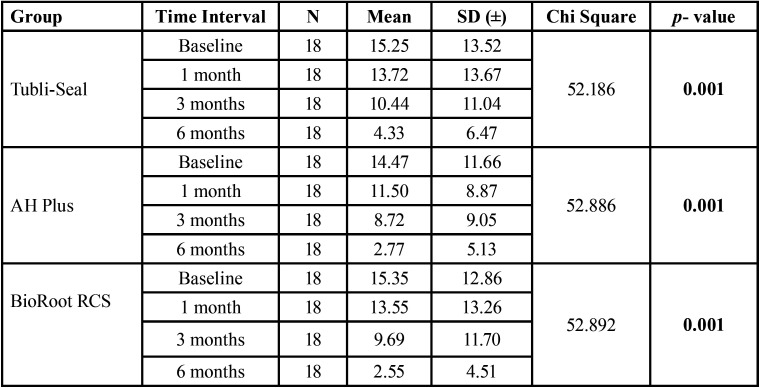
Mean size of the periapical lesion (in mm sq.) was assessed for different study groups at various time intervals (Baseline, 1 month, 3 month and 6 month). Statistical analysis using Friedman’s two-way analysis of variance showed no significant difference (*p* value < 0.05).

In Tubli-Seal group, at 24 h, 5.3% of patients showed no pain, 68.4% showed mild pain and 26.3 % showed moderate pain; at 48 h, 84.2% of patients showed no pain, 10.5% showed mild pain and 5.3 % showed moderate pain; At 72 h, 84.2% of patients showed no pain, 10.5% showed mild pain and 5.3 % showed moderate pain; At 7 d, none of the patients reported with pain. In AH Plus treated group, at 24 h 31.8% of patients showed no pain, 50% showed mild pain and 18.2 % showed moderate pain; at 48 h, 81.8% of patients showed no pain and 18.2% showed mild pain; at 72 h and 7 d, none of the patients reported with pain. In BioRoot RCS, at 24 h, 68.4% of patients showed no pain,15.8% showed mild pain while 15.8 % showed moderate pain; at 48 h, 85% of patients showed no pain and 15% showed mild pain; at 72 h and at 7 d, none of the patients had pain.

**Figure 2:**
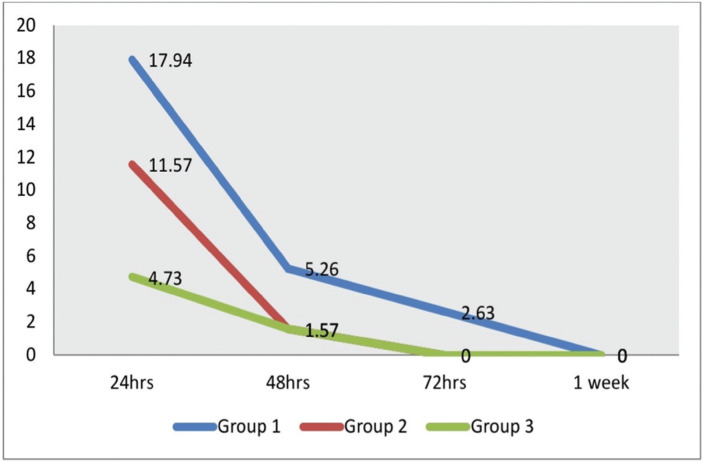
The mean postoperative pain scores were evaluated at different time intervals (24 h, 48 h, 72 h, and 7 d) using the 100mm VAS Score. (Group 1 – Tubli-Seal, Group 2 – AH Plus, Group 3 – BioRoot RCS).

The mean pain scores at 24 h, 48 h, 72 h and 7 d for Tubli-Seal treated patients are as follows; 17.94±11.35 at 24 h, 5.26±9.04 at 48 h, 2.63±7.33 at 72 h whereas none of the patients reported pain at 7 d. For the AH Plus treated patients, pain scores were seen at 11.57±11.18 at 24 h, 1.57±3.74 at 48 h whereas none of the patients reported pain at 72 h and 7 d. The BioRoot RCS treated patients is as follows; 4.73±7.72 at 24 h, 1.57 ± 3.74 at 48 h whereas none of the patients reported pain at 72 h and 7 d (Fig. 2). There was no difference in the mean pain score between the groups at any of the time intervals except for Tubli-Seal and BioRoot RCS at 24 h (*p*=0.001). In regard to sealer extrusion none of the groups showed significant difference (*p*=1.00) with sealer extrusion rate rated at 21.1% for all the test groups. There was no significant difference in the mean pain score on the basis of presence or absence of sealer extrusion except for BioRoot RCS treated patients at 24 h (*p*=0.028) and 48 h (*p*=0.040) time intervals (Table 3).

**Table 3:**
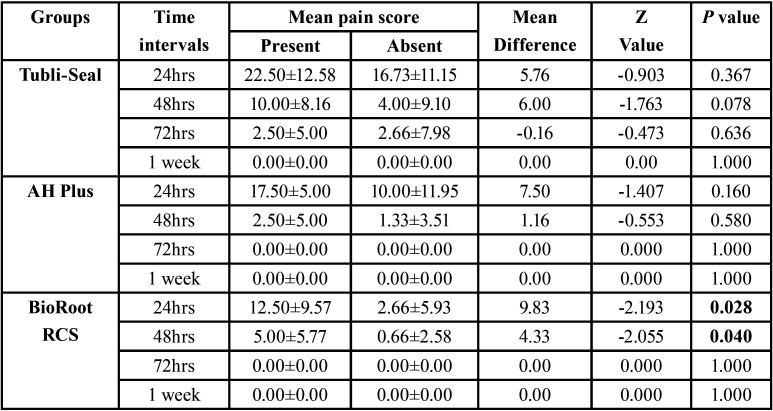
Comparison of mean pain score based on the presence of absence of extrusion within different groups. Statistical analysis using Mann Whitney test showed significance (*p*< 0.05) only at 48 h for Tubli-Seal group.

## Discussion

Bone homeostasis is disturbed in an event of apical periodontitis where an increased rate of bone resorption is witnessed. During this scenario, osteoclasts, osteoblasts, osteocytes and cementoblasts are seen to be the key cells during the process of bone formation and resorption. The null hypothesis considered for the present study was rejected since there was significant difference in the rate of periapical healing and postoperative pain levels on using different base endodontic sealers. Periodontal ligament fibroblasts synthesize and organize collagen fibers, connecting bone to the cementum, thereby repairing and regenerating the periodontal structures and aiding in periapical healing (10). On the other hand, postoperative pain after root canal treatment is considered to be a localized inflammatory reaction of the periapical tissues and is considered to be directly linked to periapical healing (11). Local inflammation due to extruded sealer in the periapical region causes postoperative pain and influences the healing process with the magnitude of inflammatory reaction seen to be influenced based on the composition of sealers (8). It is noted that on direct contact with pulpal tissues the production of reactive oxygen species is increased to seven multiples when in contact with endodontic sealers (12). It is imperative that endodontic sealers used during the root canal procedure come in direct contact with periapical tissues through the apical foramen and lateral canals, thus having a potential to affect periapical healing and post-operative pain by increasing these biomedical mediator levels.

Tubli-Seal is a zinc oxide-eugenol based sealer reported to possess increased cytotoxic and tissue-irritating potencies in *in vitro* cell culture studies and is shown to possess high cytotoxic potency and one of the oldest sealers used in endodontic practice (13). With the introduction of bioceramic based materials in endodontics significant strides have been made mainly as repair cements and root canal sealers (14). In this study, BioRoot RCS was chosen as the experimental group since there is an unavailability of data through clinical trials suggesting its superiority in promoting periapical healing or reducing post-operative pain over other endodontic sealers. BioRoot RCS is classified as a bioactive mineral root canal sealer based on innovative mineral micro-aggregate chemistry named “active biosilicate technology” (15) and is considered to be one of the most biocompatible sealers possessing osteoinductive properties in comparison to other bioceramic based sealers (16).

Endodontic therapy is known to be a complex process comprising a multitude of factors such as shaping, cleaning and obturation and thus it is very difficult to attribute the incidence of postoperative pain to any specific criteria in clinical research (17), thus there is a significant need to standardize the treatment protocol to reduce any other variable outcome. The different variables used in the present study were designed in such a manner so as to reduce as much as possible to reduce potential factors which may cause postoperative pain. Hence it was necessitated to conduct a multi visit procedure in spite of recent evidence suggesting tooth diagnosed with apical periodontitis treatment can be completed in a single visit procedure (18). The present study couldn’t take this into consideration since preoperative pain levels could have influenced the postoperative pain levels and the use of an intracanal dressing can reduce these pain score levels thus justifying the pain incidence levels by usage of endodontic sealers itself. Therefore, only when the patients who clinically exhibited no symptoms of pain or infection, obturation was carried out. The present study included only single rooted teeth for higher degree of standardization and outcome variables can be directly correlated to the treatment outcome.

The prevalence of postoperative pain recorded in this study goes in agreement with the reports estimated by Pak and White *et al*. (19) who showed that prevalence of pain after endodontic is seen to be highest at 24-48 h and reduced at only 7 d interval. The overall post-operative pain prevalence at 24 h was 63% followed by 21% at 48 h followed by 5% at 72 h and 0% at 7 d. Su *et al*. (20) had reported that postoperative pain scores post obturation were seen to be highest at 24 h to 48 h and gradually declined at 72 h, and 7 d for all the assessed groups and was the factor to consider these time frame assessments for the present study. In the present study the mean postoperative pain score was seen highest for Tubli-Seal group, followed by AH Plus and BioRoot RCS at 24 to 48 h and gradually declined at 7 d. These results could be explained by the direct cytotoxic effects of Tubli-Seal in both set and mixed state due the presence of eugenol which plays a primary role in the setting reaction. Jung *et al*. (21) had shown in their *in vitro* assessment that BioRoot RCS was seen to have least cytotoxic effect in both in premixed and set state whereas AH plus was reported to be only cytotoxic in its premixed state. Based on these reports, it can be concluded that their contact with periapical tissue will produce different inflammatory responses causing postoperative pain by the body’s innate response to increase the production of reactive oxygen species based on the leaching of different components of the sealer during the setting reaction (22).

In the present study, the mean difference in the area of the periapical lesion for Tubli-Seal, AH Plus and Tubli-Seal were 4.05, 3.86 and 6.27 respectively at 3 months and 10.22, 9.80 and 13.41 respectively at 6 months (*p*<0.05), suggesting better periapical healing with BioRoot RCS compared to AH Plus and Tubli-Seal. The results of this study can be supported by the fact that BioRoot RCS demonstrated the ability to release calcium ions (721 ppm at 3 h); B type carbonated apatite deposits were found on aged BioRoot RCS (biointeractive-related CaP-forming ability) compared to MTA Fillapex, pulp canal sealer and AH Plus sealer (23). Release of free calcium ions produces a more pronounced differentiation of macrophages and giant cells (24), leading to better reduction of microbial infection in the periapical region, subsequently promoting healing. BioRoot RCS on the other hand has shown less toxic effect on periodontal ligament cells than pulp canal sealer (zinc oxide eugenol-based sealer) as it induced higher secretion of angiogenic and osteogenic growth factors; BMP-2, VEGF, and FGF-2 (24). A recent study reported that proinflammatory cytokines such as IL-6 has been reduced and TGF-ß1 production has been increased allowing periodontal regeneration to take place (25). These mechanisms could explain the results achieved in the present study.

The periapical index (PAI) scoring was used in the present study since it gives semi-quantitative results that do not allow powerful comparison among groups (26). Thus, in this study comparison was done on the basis of the area of periapical lesion rather than the PAI scores. Measurement of area using a grid is more objective, enabling better comparability between baseline and follow-ups and reduces the chances of inter-examiner bias. The use of cone beam computed tomography is shown to accurately depict changes in cancellous bone, but an *in vivo* report has been established showing its sensitivity rate to be higher on detection of only healthy tooth but in conditions of a diseased state such as apical periodontitis the detection rate was seen to be similar to periapical radiography based on histological findings (27). Recent times have shown evolution of different CBCT systems exhibiting increased reduction in effective dosage rates in spite of this the dosage rates was seen to be 45 to 90 times higher in comparison to digital radiographic imaging and was not clinically applicable for the present study since the patients would have to go through multiple exposures for further evaluation.

Bone deposition can be considered as a clinical sign radiographically in the case of healing apical periodontitis post endodontic treatment (28). Predicting the prognosis of a tooth at the earliest, after completion of an endodontic treatment is a topic of interest from a clinical point of view. The evaluation of radiographic signs and clinical risk factors or a combination of both, could potentially hold importance in future clinical research. In the present study, the mean difference for the area of periapical lesion at baseline and 1 month was not statistically significant for any group, whereas the mean difference for the area of periapical lesion at baseline and 3 months; baseline and 6 months was statistically significant (*p* ˂ 0.05). A significant healing of periapical lesions in all the three groups at 3 months, suggests that the initial signs of the process of healing can be seen at 3 months interval. These results go in correlation with previously reported clinical study by Huumonen *et al*. reporting that three month control was adequate in establishing significant healing in cases of apical periodontitis when zinc oxide eugenol and silicone based sealers were used (29).

A 100 mm VAS scale used in the present study is considered to be an effective method for the measurement of postoperative pain in clinical research (30). Although its use has been widely reported it is considered to have certain limitations such as its subjectivity depending on individual sensitivity to pain perception. One of the methods to overcome this limitation is by a split mouth design of clinical trials since the tooth of treatment is in the same participants and potentially negates these types of errors. Periapical radiographs provide a 2-D image of a 3-D bony defect. Cone beam computed tomography (CBCT) scan of periapical healing post endodontic treatment gave similar results to that obtained by histological microscopic analysis, whereas radiographic evaluation understated the size of the periapical lesion. Cone Beam Computed Tomography (CBCT) can be used for more specific evaluation of periapical healing, limiting the field of view only to the region of interest and keeping the radiation dose at the lowest.

## Conclusions

Within the limitations of this study, the postoperative pain after root canal treatment using BioRoot RCS as endodontic sealer was less compared to AH Plus and Tubli-Seal. There was no significant difference in post-operative pain based on the extrusion of sealer except for BioRoot RCS at 48 hrs. BioRoot RCS showed better periapical healing compared to AH Plus and Tubli-Seal at 3 and 6 months. A period of 3 months was adequate to establish significant periapical healing in all the three groups. Further studies are required with other different base endodontic sealers to further justify the results obtained in this study.
